# The first successful bone marrow transplantation in Vietnam for a young Vietnamese boy with chronic granulomatous disease: a case report

**DOI:** 10.3389/fimmu.2023.1134852

**Published:** 2023-04-20

**Authors:** Binh Nguyen-Thanh, Le Nguyen-Ngoc-Quynh, Ha Dang-Thi, Chi Le-Quynh, Anh Nguyen-Thi-Van, Huyen Thuc-Thanh, Duong Dang-Anh, Pamela P. Lee, Tung Cao-Viet, Dien Tran-Minh

**Affiliations:** ^1^ Stem Cells Center, Vietnam National Children’s Hospital, Hanoi, Vietnam; ^2^ Pathophysiology and Immunology Department, Hanoi Medical University, Hanoi, Vietnam; ^3^ Department of Rheumatology, Allergy, and Immunology, Vietnam National Children’s Hospital, Hanoi, Vietnam; ^4^ Surgical Intensive Care Unit, Vietnam National Children’s Hospital, Hanoi, Vietnam; ^5^ Department of Paediatrics and Adolescent Medicine, School of Clinical Medicine, Li Ka Shing Faculty of Medicine, The University of Hong Kong, Hong Kong, Hong Kong SAR, China; ^6^ Children Heart Center, National Children’s Hospital, Hanoi, Vietnam

**Keywords:** Vietnam, case report, bone marrow transplant, Chronic granulomatous disease, inborn error of immunity

## Abstract

**Background:**

Chronic granulomatous disease (CGD) is an inborn error of immunity (IEI) disorder that results from defects in the respiratory burst activity in phagocytes, leading to the inability to kill bacterial and fungal microorganisms. CGD patients usually have a high incidence of morbidity such as infections and autoinflammatory diseases and a high mortality rate. Allogeneic bone marrow transplantation (BMT) is the only definitive cure for patients who suffer from CGD.

**Case presentation:**

We report the first transplant case of chronic granulomatous disease in Vietnam. A 25-month-old boy with X-linked CGD underwent bone marrow transplantation from his 5-year-old, full-matched human leukocyte antigen (HLA)-carrier sibling after myeloablative conditioning regimen with busulfan 5.1 mg/kg/day for 4 days, fludarabine 30 mg/m^2^/day for 5 days, and rATG (Grafalon-Fresenius) 10 mg/kg/day for 4 days. Neutrophil was engrafted on day 13 posttransplant, donor chimerism was 100% on day 30 with the dihydrorhodamine-1,2,3 (DHR 123) flow cytometric assay test that reached 38% of the normal 45 days posttransplant. Five months after transplant, the patient was free of infection with stable DHR 123 assay at 37%, and donor chimerism remained 100%. No sign of a graft-versus-host disease had been observed posttransplant.

**Conclusion:**

We suggest that bone marrow transplantation is a safe and effectual cure for CGD patients, especially for patients with HLA-identical siblings.

## 1 Introduction

Chronic granulomatous disease (CGD) is a genetically heterogeneous condition caused by defects in phagocyte nicotinamide adenine dinucleotide phosphate (NADPH) oxidase, reducing the ability to produce the superoxide anion inducing the killing of bacterial and fungal microorganisms (1–3). In patients with CGD, one of the five subunits of NADPH oxidase is detected (1). Therefore, CGD patients usually suffer from recurrent, life- threatening bacterial and fungal infections and granuloma formation (1, 2). The diagnosis is usually established early in life, although a small proportion is diagnosed during adulthood (1). Recently, six different gene mutations had been reported to involve the NADPH oxidase activation that can lead to CGD (4). Each specific mutation affects the seriousness of clinical manifestations through the remaining proportion of NADPH oxidase activity (4, 5). The specific genetic type of CGD, the presence of active infections, organ damage, and inflammatory or autoimmune complications are all factors affecting the choice of treatment protocol in a particular case (1, 2).

Allogeneic bone marrow transplantation (BMT) has been proven to be the only definitive cure for CGD until now (3, 6–8). The first bone marrow transplant was performed in 1973 (9) for a young boy with CGD, and from then, hundreds of reports have been published about stem cell transplantation for CGD (3, 7, 8). Along with the improvement in managing infections and CGD’s complications before transplant, HLA typing and donor selection, use of reduced-intensity conditioning regimens, the outcome of transplantation for CGD has been better (10, 11).

In Vietnam, lack of awareness in recognizing the clinical manifestations of different inborn errors of immunity (IEI) leads to undiagnosed IEI or late diagnosis of IEIs, including for CGD patients (5, 12, 13). Therefore, IEI patients usually suffer from severe infections and missed optimum treatment (12, 13). Here, we describe the case of the first young Vietnamese boy with X-linked CGD who achieved stable donor engraftment after bone marrow transplantation (BMT). We also describe the difficulties in clinical recognition, diagnosis, and management of CGD and other IEI diseases in a developing country such as Vietnam.

## 2 Case report

**Figure 1:**
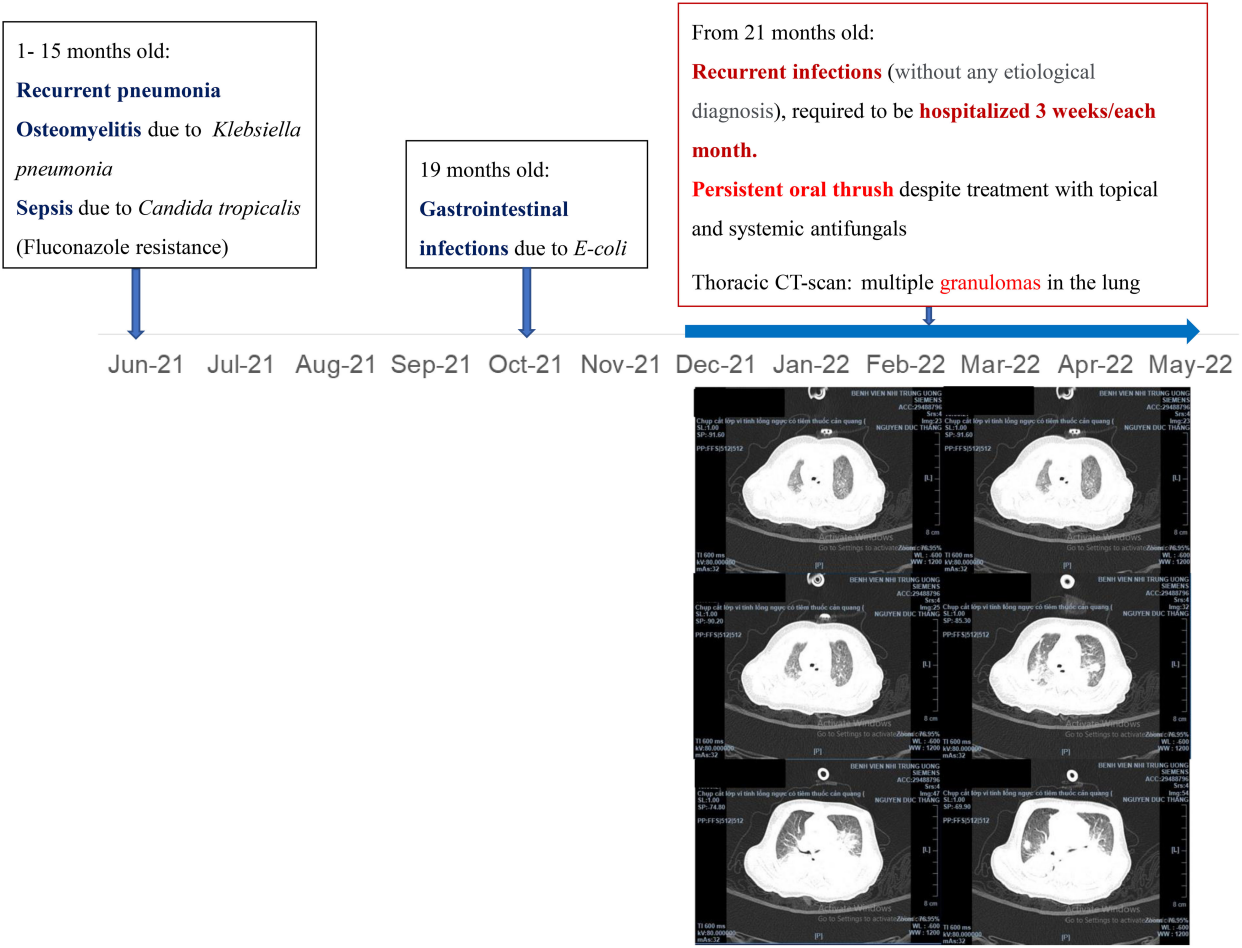
Medical history and thoracic CT-scan of the patient.

A 25-month-old boy was admitted to the Vietnam National Children’s Hospital from a local hospital with a history of 1 month of persistent high fever, progressive subacute pneumonia, severe dyspnea, and loose bloody stool. He was the second child from non-consanguineous parents, born at term gestation. There was no family history of IEI. Physical examination revealed a failure to thrive (weight-for-age < -2SD and height-for-age < -3SD of the median) without dysmorphic appearance, skin lesion, and wet rales in both lungs, and with hepatomegaly 4 cm below costal margin and splenomegaly. Complete blood counts showed high white blood cells (31x10^9^/L, normal range: 7-13x10^9^/L), with high neutrophil (18.01x10^9^/L, normal range: 2.3- 6.4x10^9^/L), normal lymphocyte (7.47x10^9^/L, normal range: 2.0-5.7x10^9^/L); normal red blood cell and high platelet (420x10^9^/L, normal range: 150-400x10^9^/L). Chest radiographs (CXR) showed bilateral perihilar haziness, while abdominal ultrasound showed hepatosplenomegaly. Thoracoabdominal computerized tomography demonstrated multiple granulomas in the lung (**Figure 1**). The serum galactomannan result was positive (subject/cut-off (S/CO) = 5.43 cut-off index (COI); normal value: < 0.5 COI); Aspergillus IgG antibody was positive at 155.55 AU/mL (normal value: < 50 AU/mL); and Aspergillus IgM antibody was positive at 350.54 AU/mL (normal value < 50 AU/mL). Blood culture and bronchoalveolar lavage fluid culture were positive with *Aspergillus fumigatus*. His medical history revealed that he had many infection episodes since he was 1-month old: recurrent and persistent pneumonia, gastrointestinal infection with bloody diarrhea, and recurrent oral thrush (**Figure 1**). Colonoscopy showed small masses (diameter 5–7 mm) in the ileum and colon. Histopathological results revealed inflammatory lesions and nuclear debris, with multiple pigmented macrophages in the lamina propria of the sigmoid colon.

**Figure 2:**
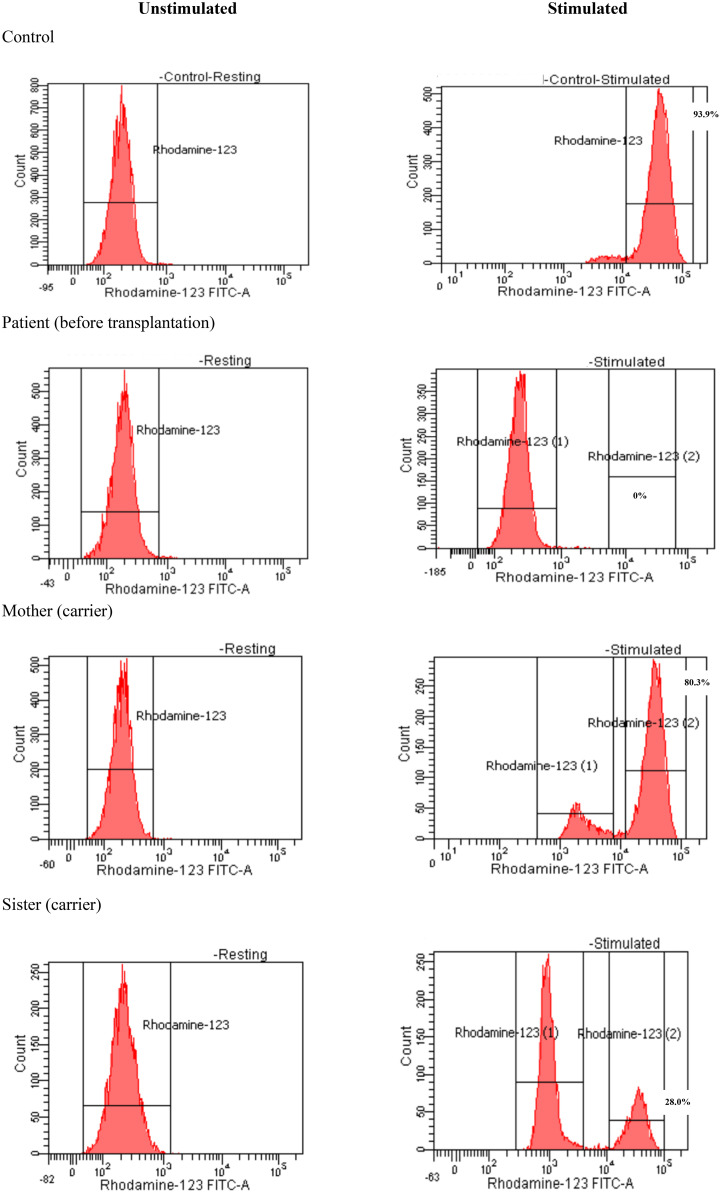
Dihydrorhodamine-1,2,3 assay of the patient and his family.

Due to unexplained severe and recurrent infection, immunological investigations have been done. Lymphocyte subsets pointed to normal T-cell counts for his age: CD3+: 5,400 cell/mm^3^ (normal range: 2,500–5,500 cell/mm^3^), CD4+: 4,000 cell/mm^3^ (normal range: 1,600–4,000 cell/mm^3^), CD8+:1,300 cell/mm^3^ (normal range: 560–1,700 cell/mm^3^); normal B-cell count (CD19+: 400 cell/mm^3^, normal range: 300–2,000 cell/mm^3^); and NKcell count (CD56+: 1,300 cell/mm^3^, normal range: 170–1,100 cell/mm^3^). The serum immunoglobulin levels were normal, with IgG: 10.2 g/L (normal range: 3.45–12.36 g/L), IgA: 1.27 g/L (normal range: 0.14–1.59 g/L), IgM: 1.48 g/L (normal range: 0.43–2.07 g/L), and normal serum IgE level (109 IU/mL, normal range: 0–230 IU/mL). The results of the dihydrorhodamine-1,2,3 (DHR 123) flow cytometric assay using the FACSCanto^®^ II (Becton Dickinson, Heidelberg, Germany), showed that the neutrophil respiratory burst was more than 99% depressed compared to control. The diagnosis of chronic granulomatous disease (**Figure 2**) had been confirmed for the patient and his family. His mother and sister had been diagnosed as X-linked CGD carriers, with oxidase-positive cells being 80.3% and 28.0%, respectively (**Figure 2**). Whole exome sequencing was performed on the Illumina sequencing machine (Illumina, CA, USA) for the identified heterozygous mutation in the *CYBB* gene (c.867G>A; p. Trp289*). Unfortunately, gp91^phox^ protein expression could not be done in our country. This X-linked mutation has been confirmed in his mother and sister as a hemizygous carrier. The patient was treated with amphotericin B (lipid complex) for 4 weeks to manage *Aspergillus fumigatus* until the serum galactomannan test was negative and treatment was switched to prophylactic voriconazole 5 mg/kg oral daily, combined with prophylactic cotrimoxazole-trimethoprim. After 5 months, voriconazole was replaced by micafungin before transplantation to avoid drug interaction between azoles and busulfan levels. The patient received oral prednisolone 1 mg/kg/day for 1 month and intake was tapered gradually over 3 months to resolve granulomata in the lung and intestinal tract. Nonetheless, the patient had poor response to these treatments. Repeated chest CT-scans and colonoscopy results showed that the lesions had not changed much after conventional treatment.

**Figure 3:**
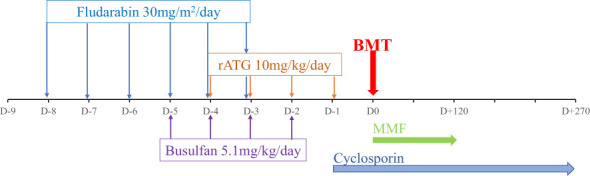
Conditioning regimen and Graft versus Host disease (GvHD) prophylaxis of the patient.

**Figure 4:**
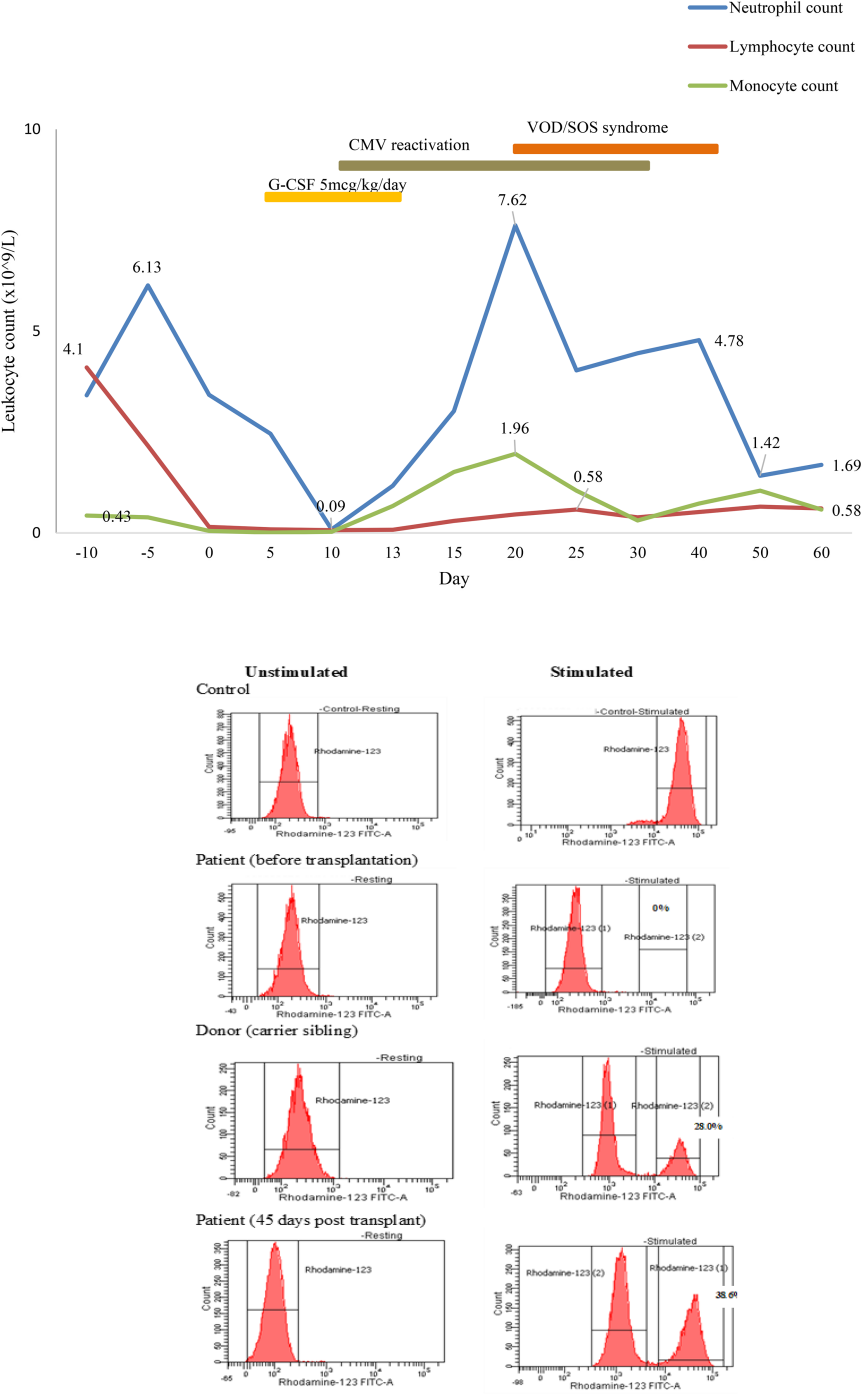
Engraftment of the neutrophil posttransplant.

Subsequently, he was referred to the BMT team when he was 31-months old and his body weight reached 9 kg without major infection. Low resolution human leukocyte antigen (HLA) typing from his older sister indicated a full-matched HLA typing in HLA class I loci (HLA-A: 02, 29; HLA-B: 15, 40; HLA-C: 08, 08) and class II loci (HLA-DR: 09, 12). The matched HLA 5-year-old sister, who weighed 15.6 kg at that time, was chosen as the donor despite being a hemizygous CGD carrier with 30% of neutrophils displaying neutrophil respiratory burst. The donor and recipient were of the same blood type, O+. According to the European Society for Blood and Marrow Transplantation/European Society for Immunodeficiencies (EBMT/ESID) Inborn Errors Working Party (IEWP) (14), myeloablative conditioning included fludarabine 30 mg/m^2^ i.v. once a day from day -8 to day -3, busulfan i.v. 5.1 mg/kg daily from day -5 for 4 days, divided into four doses, and ATG-Fresenius (Grafalon, Neovil Biotech, Waltham, MA, USA) i.v. 10 mg/kg for 4 days from day -4 (**Figure 3**). Graft-versus-host disease prophylaxis consisted of cyclosporine 2.4 mg/kg/day i.v. for 21 days and then switched to oral as soon as the patient could consume orally. Mycophenolat mofetil 1,200 mg/m^2^ p.o. daily was also given (**Figure 3**). Stem cells were harvested from the donor’s bone marrow and infused to the recipient with a dose of 2.96 × 10^8^ nucleated cells, 7.94 × 10^6^/kg CD34^+^ cells of recipient body weight. G-CSF i.v. at 5 mg/kg was given daily from day 5 until neutrophil engraftment. The patient had been continuously given intravenous antimicrobials for managing lung and gut infections, and cotrimoxazole for *Pneumocystis jirovecii* prophylaxis. He was also given intravenous immunoglobulin for infection prophylaxis. Neutrophil engraftment (defined as ANC >0.5 × 10^9^/L) appeared on day 13 posttransplant (**Figure 4**). Red blood cells engrafted around day 25 posttransplant, and platelet increased to >20 G/L 36 days posttransplant. Cytomegalovirus (CMV) reactivated on day 12 posttransplant and the patient was given intravenous ganciclovir for 21 days. The patient also developed features of mild/moderate sinusoidal obstruction syndrome (SOS) according to the EBMT criteria on day 20, including weight gain of >10% above the baseline value, tender hepatomegaly, and ascites, with the highest serum bilirubin of 32.8 μmol/L (normal range: 2–8 μmol/L) (15). Abdominal ultrasound revealed hepatomegaly, ascites, normal triphasic hepatic venous flow, and no sign of portal vein dilation. He was treated with defibrotide 6.25 mg/kg intravenously every 6 h (25 mg/kg/day on total daily dose) for 21 days and other supportive management. No sign of acute Graft versus Host disease (aGvHD) was observed post-BMT. Whole peripheral blood chimerism showed 100% of donor chimerism 30 days after bone marrow transplantation. The DHR 123 test result, as assessed on flow cytometry, was 38% at 45 days posttransplant (**Figure 4**). He was infection-free at 2 months posttransplant and was discharged. At home, the patient had been maintained on oral ciprofloxacin and oral voriconazole until 5 months posttransplant. Whole blood chimerism 5 months after transplant remained 100% of the donor, with a stable DHR 123 assay on flow cytometry at 37%. Problems with bowel movement and hematochezia disappeared after 4 months, but colonoscopy has not been repeated yet due to absence of consent from the family.

## 3 Discussion

Here, we report the first successful bone marrow transplantation for a young boy with CGD in Vietnam. CGD is a genetically heterogeneous condition caused by defects in oxidative metabolism in phagocytes (1, 2, 16). Pneumonia, liver abscess, lymphadenitis, and skin infection are the most frequent infections involved in CGD (16, 17). Patients with CGD are prone to granulomata, chronic pulmonary disease, and autoimmunity (16, 18, 19). Inflammatory bowel disease is common in CGD, although not as severe as a typical Crohn’s disease (19). Lung infection is the most common disorder, and patients with CGD can develop a characteristic syndrome of dyspnea, hypoxia, and fever leading to respiratory failure and death within 10 days after inhalation of large burdens of fungal spores and hyphae (20). In our case report, the patient had clinical presentations since he was 1-month old, with infections in his skin, bone, and lung due to bacterial and fungal, and gastrointestinal symptoms suspected very early-onset inflammatory bowel disease. Unfortunately, he had not been diagnosed with CGD until he was 25-months old after many infections and complications.

The gene *CYBB* is located at chromosome Xp21.1 encoding gp91^phox^, which is the enzymatic center of the NADPH oxidase (4, 5, 16). Five other autosomal recessive etiologies of CGD have been described, namely, CYBA, NCF1, NCF2, NCF4 and CYBC (4, 5). X-linked CGD that has been caused by a *CYBB* mutation accounts for approximately 60% to 70% of CGD cases (4), and CGD occurs more often in men than in women. Young boys with X-linked CGD seem to have earlier manifestation and severe prognosis (16). In consequence, they should receive more intensive care and treatment. Our patient had 0% of normal oxidase-positive neutrophils with serious early-onset infections and he belonged to the high-risk CGD group (**Figures 1**, **2**).

Oral trimethoprim-sulfamethoxazole for antibacterial prophylaxis and antifungal prophylaxis with itraconazole are the lifelong treatment for children with CGD (1, 2, 16). Despite the improvement in managing infections and inflammatory complications, mortality remains high through the fourth decade of life (21). Bone marrow transplantation has been used successfully to clear refractory infections for patients with CGD, and the outcome is especially better in younger patients before they develop any unrepairable organ dysfunction (7, 22, 23).

Myeloablative busulfan-based conditioning regimen has been applied for this case following the IEWP/EBMT guidelines (**Figure 3**) (14). This conditioning regimen with HLA-matched bone marrow donors has been proven to be effective in donor engraftment on allogeneic transplantation in CGD (6). However, the main toxicity effect of busulfan on endothelial cells was concentration- dependent. High busulfan dose according to myeloablative conditioning could increase incidence of hepatotoxicity, including sinusoidal obstruction syndrome (SOS) (15, 24). Our patient suffered from moderate SOS and has been treated successfully with defibrotide. Consequently, some recent studies disclosed successful reduced-intensity conditioning regimen on overall survival and event-free survival after transplantation in CGD (7, 8, 10, 25). The reduced-intensity conditioning requires laboratory measurement of busulfan serum levels and determination of the area under the concentration curve, which is unavailable in Vietnam. Hence, in future, busulfan dose regimen and therapeutic drug monitoring may be needed to control the risk of SOS complication.

Full-matched HLA carrier sibling was chosen as the donor because there was no other available matched donor. DHR 123 assay exhibited a mosaic pattern for normal oxidase-positive neutrophils in the mother and sister (80.3% and 28.0%, respectively). The X chromosome inactivation (lyonization) in women explains this phenomenon (26). Lyonization refers to the normal process by which one of the X chromosomes is inactivated randomly in somatic cells since early fetus development. After transplant, 38% of the recipient’s neutrophils displayed normal phagocyte respiratory burst at DHR 123 flow cytometry (**Figure 4**). This mixture of NADPH-oxidase producing and non-producing cells was according to the degree of lyonization in the X-linked carrier sibling donor and X-linked mother. In the recipient, X chromosome inactivation may be activated after engraftment. The question is how many sufficient myeloid cells from the donor are necessary to cure CGD. Data from Connelly et al. suggested that patients with donor myeloid chimerism above 20% might be adequate for the lifelong prevention of infection and inflammation (22). Nevertheless, additional studies are required to find out whether mixed chimerism is sufficient to cure the disease phenotype and whether it is associated with inflammatory and autoimmune manifestations (16, 22). Therefore, prolonged clinical follow-up, repeated DHR 123 assay, and donor chimerism at least 9 to 12 months posttransplantation are required to confirm efficacy in ameliorating CGD-related diseases in this case.

Vietnam has a population of 99.5 million (from the statistics of 2022) (27), and children under 5 years of age are about 7.61 million. IEIs had been diagnosed and recorded in Vietnam since 2011 (28). Up to 2010, fundamental immunological techniques had not been done regularly, and genetic analyses were performed abroad due to a shortage of local clinical immunologists and laboratory centers. Since 2015, with support from an international clinical immunologist, the awareness of IEIs has been raised and the number of diagnosed IEI cases increased. BMT for children in Vietnam was first conducted in 2000, including allogeneic transplantation for thalassemia, aplastic anemia, leukemia patients, and autologous transplantation for neuroblastoma. However, for patients with IEIs, potential for BMT was not available, and most of the patients usually died before BMT is considered. The first IEI transplantation was done in Vietnam National Children’s Hospital for a severe combined immunodeficiency disease (SCID) patient in 2014. Our patient was 31-months old during transplantation, and 2 months posttransplant, he was discharged from the hospital without infection. Until now, Vietnam National Children’s Hospital is the only center in the whole country that supports BMT for IEI patients.

Our report emphasized the increasing prevalence of IEIs in Vietnam. More and more cases are being diagnosed, subsequently increasing awareness among pediatrics and the community, and as such, modern medical diagnostic equipment are needed. Consequently, BMT is the only definitive cure for patients with IEIs, preferably before developing serious infections and organ impairment because of complications.

## 4 Conclusion

In summary, bone marrow transplantation provided an effective cure for our CGD patient. This cure could be contemplated in all CGD patients who have a full- matched HLA-typing donor and who tolerate critical infections and complications despite prophylactic management. Myeloablative busulfan-based conditioning regimen has been shown to reach acceptable engraftment.

## Data availability statement

The original contributions presented in the study are included in the article/supplementary material. Further inquiries can be directed to the corresponding author.

## Ethics statement

The studies involving human participants were reviewed and approved by Institutional Review Board of Vietnam National Children’s Hospital. Written informed consent to participate in this study was provided by the participants’ legal guardian/next of kin. Written informed consent was obtained from the patient’s parent, for the publication of any potentially identifiable images or data included in this article.

## Author contributions

BN-T, LN-N-Q, HD-T, CL-Q, AN-T-V, HT-T, DD-A, TC-V, DT-M, and PL contributed to the conception and design of the study. LN-N-Q wrote the first draft of the manuscript. BN-T, LN-N-Q, and HD-T collected the data and wrote sections of the manuscript. All authors contributed to the manuscript revision and read and approved the submitted version.
